# Gene-expression analysis of adult-onset Still’s disease and systemic juvenile idiopathic arthritis is consistent with a continuum of a single disease entity

**DOI:** 10.1186/s12969-015-0047-3

**Published:** 2015-11-20

**Authors:** Nanguneri Nirmala, Arndt Brachat, Eugen Feist, Norbert Blank, Christof Specker, Matthias Witt, Jan Zernicke, Alberto Martini, Guido Junge

**Affiliations:** Novartis Institutes for BioMedical Research, Inc., 45 Sidney Street, Cambridge, MA 02139 USA; Novartis Institutes for Biomedical Research, CH-4002 Basel, Switzerland; Department of Rheumatology and Clinical Immunology, Charité- Universitätsmedizin, Chariteplatz 1, 10117 Berlin, Germany; Division of Rheumatology, Department of Medicine, University of Heidelberg, Im Neuenheimer Feld 410, D-69120 Heidelberg, Germany; Klinik für Rheumatologie & Klinische Immunologie, SJK – University Hospital Essen, Propsteistrasse 2, 45239 Essen, Germany; Division of Rheumatology and Clinical Immunology, Med. Klinik and Poliklinik IV, University of Munich, Pettenkoferstrasse 8a, 80336 Munich, Germany; University of Genoa and G Gaslini Institute, Head Pediatria II, Reumatologia, IRCCS G. Gaslini, Largo G. Gaslini 5, 616147 Genoa, Italy; Novartis Pharma AG, CH-4002 Basel, Switzerland

**Keywords:** Adult-onset Still’s disease, Canakinumab, Gene expression, Interleukin-1β, Systemic juvenile idiopathic arthritis

## Abstract

**Background:**

Adult-onset Still’s disease (AOSD), a rare autoinflammatory disorder, resembles systemic juvenile idiopathic arthritis (SJIA). The superimposable systemic clinical features of AOSD and SJIA suggest both clinical phenotypes represent the same disease continuum with different ages of onset. To further characterize the similarity between AOSD and SJIA at the molecular level, 2 previously identified response gene sets in SJIA were used to investigate how genes that respond to interleukin (IL)-1β inhibition with canakinumab in SJIA patients behave in AOSD patients with active disease prior to IL-1β targeting therapy, relative to healthy subjects.

**Findings:**

All genes downregulated in SJIA patients following canakinumab treatment were upregulated in most patients with active AOSD prior to canakinumab treatment, relative to healthy subjects. A few patients with milder AOSD had expectedly gene-expression patterns that resembled those in healthy subjects. Comparison of the gene-expression patterns with neutrophil counts showed a correlation between elevated neutrophil numbers and upregulation of canakinumab-responsive genes. Correspondingly, most genes upregulated following canakinumab treatment in patients with SJIA patients were downregulated in the majority of AOSD patients.

**Conclusions:**

These results further support the concept of a Still’s disease continuum that includes both a pediatric/juvenile onset (SJIA) and adult onset (AOSD) form.

## Findings

### Background and research hypothesis

Adult-onset Still’s disease (AOSD) is a rare autoinflammatory disorder resembling the pediatric syndrome systemic juvenile idiopathic arthritis (SJIA) [1, 2]. Patients with either AOSD or SJIA exhibit classical clinical and laboratory features, including daily spiking fever, arthralgia or arthritis, evanescent rash, and increased white blood cell count (mainly neutrophils) [1, 3–5]. Because of the superimposable systemic clinical features of SJIA and AOSD, it has been suggested that these clinical phenotypes represent the same disease continuum with different ages of onset. It has, therefore, been proposed that patients with the systemic features of SJIA but without arthritis be classified as having Still’s disease based on analogy with AOSD [2]. Of note, when children were first described in the 19^th^ century with what is now called “SJIA,” the term “Still’s disease” was used [6].

In addition to having a similar clinical picture, evidence suggests that AOSD and SJIA are also comparable on a molecular level. Both conditions are characterized by activation of the innate immune system, including elevations of inflammatory cytokines and proteins such as interleukin (IL)-1, IL-6, IL-18, and S100 proteins [7–11]. Moreover, patients with AOSD or SJIA can experience macrophage activation syndrome, a severe, potentially life-threatening complication [12, 13].

The most compelling evidence demonstrating that AOSD and SJIA largely share the same pathophysiology may be that both are highly responsive to IL-1 inhibition. Translational and clinical studies suggest that the proinflammatory cytokine IL-1 plays a pivotal role in the pathogenesis of SJIA. Serum samples from patients with SJIA have been shown to induce the transcription of innate immunity genes, including IL-1, in healthy peripheral blood mononuclear cells [14]. In addition, these cells in patients with SJIA demonstrated a possible IL-1–driven signature [15]. More importantly, the pivotal role of IL-1 in the pathogenesis of SJIA has been validated by the clinical efficacy of canakinumab — a high-affinity human monoclonal anti − IL-1β antibody — in phase 2 and 3 SJIA trials [16, 17]. Evidence for the clinical efficacy of IL-1 inhibition in adult patients with moderate to severe AOSD, including those resistant to standard therapy, is principally derived from small observational studies [10, 18–22]. Further, results from 1 open-label, randomized trial suggest that IL-1 inhibitor therapy is effective in AOSD and may minimize or avoid the need for glucocorticoids in most patients [23, 24].

Analyses of gene-expression profiles can be clinically useful not only for disease classification, diagnosis, and prognosis but also to identify disease-specific treatment effects that target underlying pathologic mechanisms. In a previous gene-expression analysis using blood samples from patients with SJIA treated with canakinumab in phase 3 trials, canakinumab strongly repressed many inflammation- and innate-immunity–related genes, including those in the IL-1–signaling pathways, in canakinumab responders [25]. To evaluate if AOSD is also an IL-1 − driven condition, the present study investigated how genes that respond to IL-1β inhibition with canakinumab in patients with SJIA behave in patients with active AOSD, relative to healthy subjects. It was hypothesized that because AOSD and SJIA are closely related conditions, genes that respond to canakinumab treatment in patients with SJIA may be inversely dysregulated in patients with AOSD prior to anti − IL-1β therapy. If this proved to be the case, then a similar set of genes in AOSD should be responsive to anti − IL1-β therapy.

### Patients and methods

In the previous SJIA gene-expression analysis, pre- and post-canakinumab treatment blood samples were obtained from patients participating in two phase 3 clinical trials (β-SPECIFIC-1 and β-SPECIFIC-2) [25]. To identify canakinumab-responsive genes, baseline (pretreatment) and day-3 (post-treatment) samples were compared from patients who clearly benefited from canakinumab treatment by achieving a ≥50 % improvement by adapted pediatric American College of Rheumatology criteria at day 15 (median age, 9 years). The canakinumab-response signature in SJIA included genes that were significantly differentially expressed (paired *t*-test: *P* <.05; ≥1.5-fold differential expression) at day 3 compared with baseline, represented by 577 downregulated probe sets and 728 upregulated probe sets.

For the present gene-expression analysis in AOSD, blood samples were obtained from 17 patients (median age, 37 years; 59 %, female) under investigation for the efficacy of canakinumab in patients with active AOSD [NCT02204293] [26]. Blood samples were also obtained from 19 healthy subjects included in the control group (median age, 26 years; 79 %, female). The probe sets identified in the blood samples of patients with SJIA were used for supervised visualization of gene-expression values in the untreated patients with AOSD and healthy subjects. The data were median-centered per gene to visualize the direction of differential expression more clearly.

Whole blood samples were collected in PAXgene Blood RNA tubes (Qiagen, Hilden, Germany) and stored at −80 °C. Total RNA was subsequently isolated with the PAXgene Blood RNA Kit (Qiagen). The synthesis of cDNA was performed using the Ovation® RNA Amplification System V2 including the Ribo-SPIA® amplification process according to the instructions of the manufacturer (NuGEN Technologies Inc., San Carlos, CA). The amplification process was performed in 3 stages: (1) a 1st-strand cDNA synthesis with oligo(dT) primers and Ovation WB Reagent (NuGEN), (2) a 2nd-strand cDNA synthesis, and (3) a single-primer, linear isothermal amplification (SPIA™, NuGEN) that produced amplified single-stranded biotin-labeled cDNA. The cDNA was hybridized to GeneChip® Human Genome U133 Plus 2.0 Array as specified by the manufacturer (Affymetrix, Inc., Santa Clara, CA). Gene-expression values were stored in CEL files that were used for robust multi-array average normalization with the *affy* and *gcrma* R packages. Normalized data were then scaled to a trimmed mean value of 150. The significance of gene-set enrichment was estimated using the ROAST method as implemented in R, [27] applying 10,000 rotations to the data set.

## Results and discussion

**Fig. 1:**
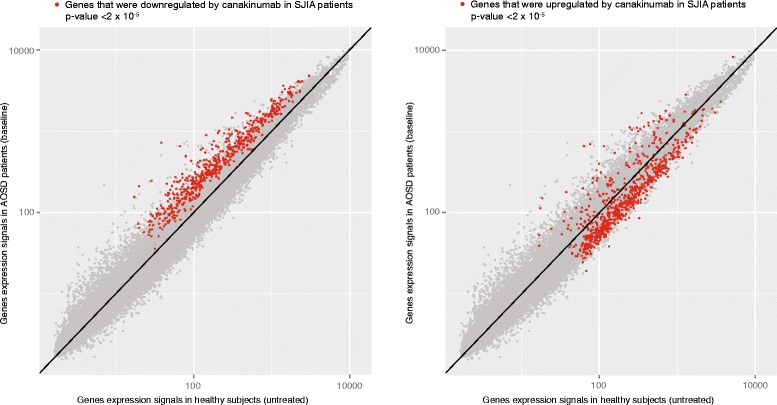
Comparison of gene-expression data for patients with AOSD prior to canakinumab treatment and healthy subjects. *Dots* show expression levels of individual genes where the average value in healthy subjects is shown on *x axis* and the average value in patients with AOSD is shown on *y axis*. Genes represented in *red* are those previously found to respond to canakinumab treatment in patients with SJIA. Genes downregulated by canakinumab in patients with SJIA were upregulated in untreated patients with AOSD, relative to healthy subjects (*plot on left*). Correspondingly, most genes previously found to be upregulated by canakinumab in SJIA were downregulated in untreated patients with AOSD (*plot on right*). *P* values refer to significance of differential expression of canakinumab gene sets

**Fig. 2:**
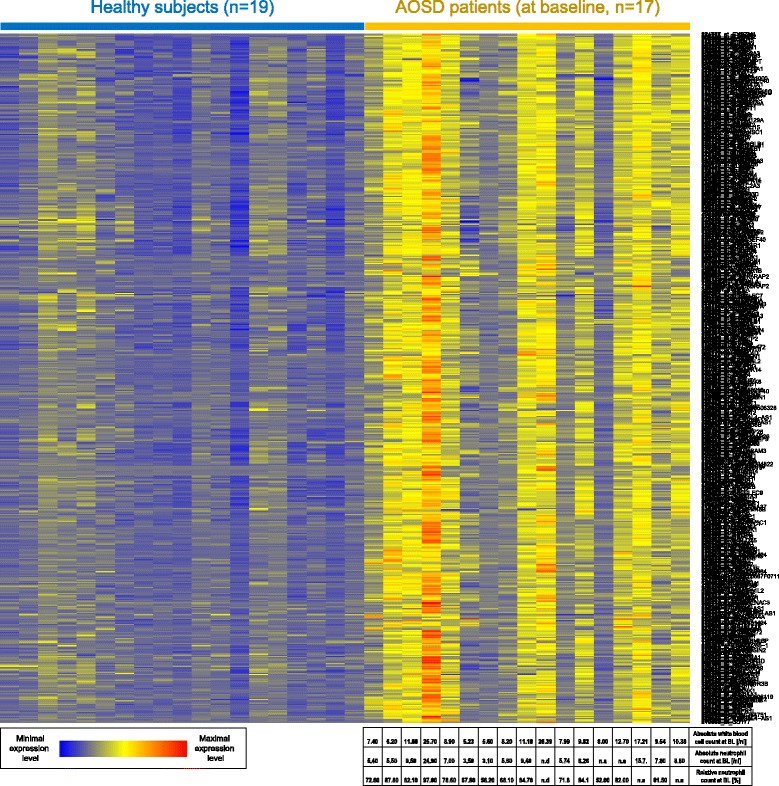
Color-coded expression values of genes ***down*** regulated following canakinumab treatment in patients with SJIA. Gene-expression values are shown for healthy subjects and patients with AOSD prior to canakinumab treatment. Transcripts shown in *rows* and patient samples in *columns*. For patients with AOSD, baseline (BL) blood cell counts are also provided (*bottom*)

**Fig. 3:**
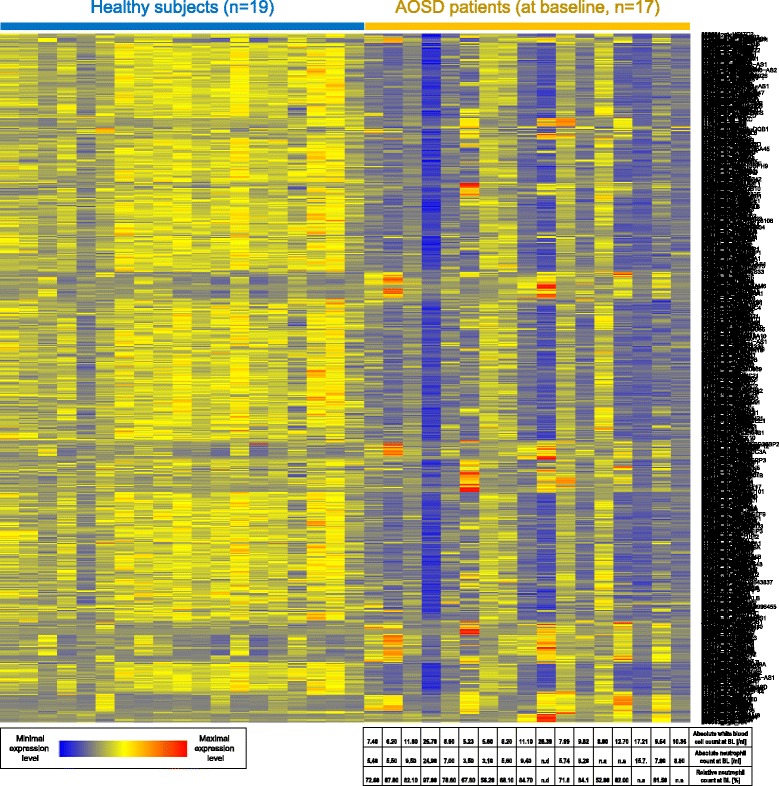
Color-coded expression values of genes ***up*** regulated following canakinumab treatment in patients with SJIA. Gene-expression values are shown for healthy subjects and patients with AOSD prior to canakinumab treatment. Transcripts shown in *rows* and patient samples in *columns.* For patients with AOSD, baseline (BL) blood cell counts are also provided (*bottom*)

The behavior of canakinumab responsive genes in patients with AOSD and healthy subjects is shown in Figs. 1, 2, and 3. Figure 1 displays the average expression values in the AOSD and healthy groups, whereas Figs. 2 and 3 show the relative expression values in all individuals separately. All genes that were downregulated following canakinumab treatment in patients with SJIA showed upregulation in most patients with AOSD, relative to healthy subjects (Figs. 1 and 2). These upregulated genes included various genes related to innate immunity, including several members of the IL-1–signaling pathways, e.g. IL-1β, IL-1RAP, IL-1RN, IL-1R1, and IL-1R2. A few patients with milder AOSD had gene-expression profiles that rather resembled those of the healthy subjects (Fig. 2). Comparison of the AOSD gene-expression patterns with neutrophil counts showed that upregulation of IL-1 − associated gene expression was particularly pronounced in patients with strongly elevated neutrophil numbers and that patients with comparatively low neutrophil counts showed expression of canakinumab responsive genes at levels similar to healthy subjects. Correspondingly, most of the genes that were found to be upregulated following canakinumab treatment in patients with SJIA showed downregulation in most AOSD patients (Figs. 1 and 3), with the transcriptional patterns slightly more heterogeneous. These genes included many regulators of proliferation and immune-cell activity, such as AKT3, CD24, CD28, CD3D, CD6, CD69, CDC25B, and CDC7.

While relatively new, gene expression allows analysis of hundreds and thousands of different genes at a given time instead of a handful of candidates, and represents the most advanced and comprehensive approach to screening gene activity as well as molecular networks involved in the innate immune system. Nonetheless, SJIA and AOSD are rare autoinflammatory conditions; therefore, large prospective and longitudinal trials are difficult to generate which explains the relatively small sample size of this analysis. Disease duration and severity, prior treatment with other biologics and concomitant corticosteroid exposure during the trial may have influenced the SJIA findings. With regard to the AOSD baseline analysis it should be noted that future analysis needs to confirm the hypothesized treatment response and downregulation of canakinumab responsive genes.

In conclusion, the results of this gene-expression analysis are consistent with and further support the concept of a Still’s disease continuum that includes both a pediatric/juvenile onset (SJIA) and adult onset (AOSD). These findings support further clinical evaluation of anti − IL-1β therapy in patients with AOSD given that AOSD appears to be an IL-1–driven condition similar to SJIA.

### Ethical approval

Study NCT02204293 (Canakinumab for Treatment of Adult Onset Still’s Disease) was approved by the Paul-Ehrlich-Institut (PEI) Germany (Postfach D-63207 Langen, Germany) on April 3, 2012 and the Ethic Committee Landesamt für Gesundheit und Soziales (LAGeSo) (Friedrichstrasse 16 in D-10969 Berlin, Germany).

## Abbreviations

AOSD: adult-onset Still’s disease
BL: baseline
IL: interleukin
SJIA: systemic juvenile idiopathic arthritis.
